# Advances in Molecular Techniques for Detecting Sweet Potato (*Ipomoea batatas* (L.) Lam) Viruses: A Comprehensive Review

**DOI:** 10.3390/v18080908

**Published:** 2026-08-18

**Authors:** Muhammad Abul Kalam Azad, Nanziba Ibnat, Saleh Shafique Chowdhury, Saaimatul Huq, Shahidul Islam

**Affiliations:** 1 Department of Agriculture/Agricultural Regulations, University of Arkansas at Pine Bluff, 1200 North University Dr., 148 Woodard Hall, Mail Slot 4913, Pine Bluff, AR 71601, USA; ibnatn5421@uapb.edu (N.I.); shafiqs5419@uapb.edu (S.S.C.); 2 Department of Biochemistry & Molecular Biology, Winthrop P. Rockefeller Cancer Institute, University of Arkansas for Medical Sciences (UAMS), 449 Jack Stephens Dr., Little Rock, AR 72205, USA; shuq@uams.edu

**Keywords:** sweet potato virus, molecular diagnostic techniques, ELISA, qPCR, CRISPR/Cas-based virus detection, sweet potato feathery mottle virus (SPFMV)

## Abstract

Sweet potato (*Ipomoea batatas* (L.) Lam) is an important global food crop, but its production is threatened by numerous viral pathogens. More than 30 RNA and DNA viruses have been reported worldwide, making rapid and accurate detection essential for disease management, epidemiological surveillance, germplasm exchange, and resistance breeding. Although previous reviews have addressed sweet potato viruses and individual diagnostic methods, a comprehensive synthesis of emerging molecular technologies remains limited. This review addresses that gap by critically integrating recent advances from PCR-based and isothermal assays to high-throughput sequencing, CRISPR-based diagnostics, biosensors, nanotechnology, and artificial intelligence-driven detection platforms. Conventional approaches, including symptom observation, biological indexing, electron microscopy, and ELISA, have contributed to early virus identification but often lack the sensitivity, specificity, and speed needed for modern diagnostics. Molecular and isothermal techniques have substantially improved detection accuracy and enabled rapid identification and field-deployable diagnostics of diverse and mixed infections, while sequencing, CRISPR, biosensors, and AI-based platforms offer greater capacity for detecting novel and emerging viruses. This review discusses the comparative evaluation of molecular technologies for sweet potato virus detection in terms of diagnostic performance, cost-effectiveness, speed, and suitability for both laboratory and field applications, while highlighting future priorities for next-generation virus diagnostics. Integrating portable and high-throughput diagnostic platforms will strengthen virus surveillance, support virus-free planting material production, and promote sustainable sweet potato production worldwide.

## 1. Introduction

Sweet potato (*Ipomoea batatas* (L.) Lam) is a short-day crop that are highly adaptable and resilient to stress, ranking among the world’s ten most important food crops [1]. The global sweet potato production was estimated at about 52 million tons in 2021, according to the Food and Agriculture Organization (FAO) [2]. Additionally, national production has also increased steadily, rising from 36,000 tons in 1994 to 59,700 tons in 2023 [3]. It is grown in more than 120 countries, where it serves not only as a major source of carbohydrates, vitamins, dietary fiber, and carotenoids, but also as a source of antioxidants, anticancer, and antimicrobial sources, particularly for resource-limited populations [1,4,5]. In addition, sweet potato plays an important role in improving food security due to its short growth cycle and high yield potential. It also supports smallholder farmers’ income and contributes to agricultural resilience and environmental sustainability because it can grow well in poor soils and under changing climatic conditions [3,6].

However, sustainable sweet potato production is threatened by continuous monoculture, misuse of pesticides, and the large-scale movement and mechanized cultivation of sweet potatoes have accelerated the spread of diseases, particularly viral infections [7]. Many sweet potato plants are repeatedly infected by several viruses, causing average yield losses of 20–40% [8]. Even in Africa, orange-fleshed varieties rich in pro-vitamin A have been introduced to combat nutritional deficiencies, still viral diseases have severely hampered their adoption [9]. Sweet potato virus disease (SPVD) is the most damaging viral disease triggered by the co-infection of two viruses: sweet potato chlorotic stunt virus (SPCSV), transmitted by whiteflies, and sweet potato feathery mottle virus (SPFMV), transmitted by aphids. This combined infection is more detrimental than individual viral infections, reducing tuber yield by up to 85% [10,11]. On the other hand, sweet potato leaf curl virus (SPLCV), a *Begomovirus*, belonging to the family *Geminiviridae*, has recently been identified as the third most common virus infecting sweet potato and can also show synergistic interactions with SPCSV [12].

As sweet potato propagated via vines, root slips, and tubers, which makes it inherently vulnerable to progressive accumulation of viral infections across planting seasons [13]. Because most individual virus infections are either asymptomatic or produce only mild, non-specific signs, virus-infected propagation materials can circulate undetected in seed systems, worsening disease spread [10,14]. Also, in many countries farmers often recycle planting material from their own fields each year. As a result, viruses present in infected plants are easily carried over to newly planted fields, leading to lowered yields [15].

Therefore, understanding sweet potato diseases that occur during growth, harvest, and storage is important for quick and perfect diagnosis. Effective disease management is necessary to maintain high yields and ensure the quality and safety of sweet potatoes. Additionally, accurate detection and identification of sweet potato viruses are also crucial for producing virus-free planting materials, supporting plant quarantine and safe germplasm exchange, epidemiological surveillance, and developing resistant varieties through breeding programs [10,16]. Since the early 2000s, the adoption of molecular methods has greatly improved with the understanding of composition, biology, and management of sweet potato virus complexes [10,17]. This helps the transformation of sweet potato virus diagnostics rapidly from conventional serology and polymerase chain reaction (PCR) toward faster, more sensitive, and field-deployable molecular tools. RT-PCR remains the most widely used and reliable method for detecting RNA viruses in sweet potato due to its high sensitivity and specificity [18]. Other techniques such as nested PCR, immune capture PCR, Loop-mediated isothermal amplification (LAMP), and qPCR are also used to improve detection efficiency and reduce false negatives.

Despite significant progress in sweet potato virus diagnostics, several technological gaps remain. Most conventional and PCR-based methods rely on prior knowledge of the target virus and are often limited in detecting highly divergent, emerging, or mixed viral infections that commonly occur in sweet potato [16,17]. In addition, the high cost of equipment, dependence on laboratory infrastructure, lengthy sample processing, and limited field applicability restrict the widespread adoption of many advanced diagnostic approaches, particularly in resource-limited regions where sweet potato is an important food-security crop. Emerging technologies such as high-throughput sequencing (HTS), CRISPR/Cas-based diagnostics, portable isothermal amplification platforms, biosensors, and artificial intelligence-assisted detection offer opportunities to overcome these limitations through improved sensitivity, specificity, multiplex detection, real-time analysis, and field deployment. Therefore, integrating these complementary technologies is essential for developing rapid, accurate, affordable, and scalable diagnostic systems capable of supporting effective virus surveillance, certification of virus-free planting materials, and timely disease management strategies.

**Figure 1:**
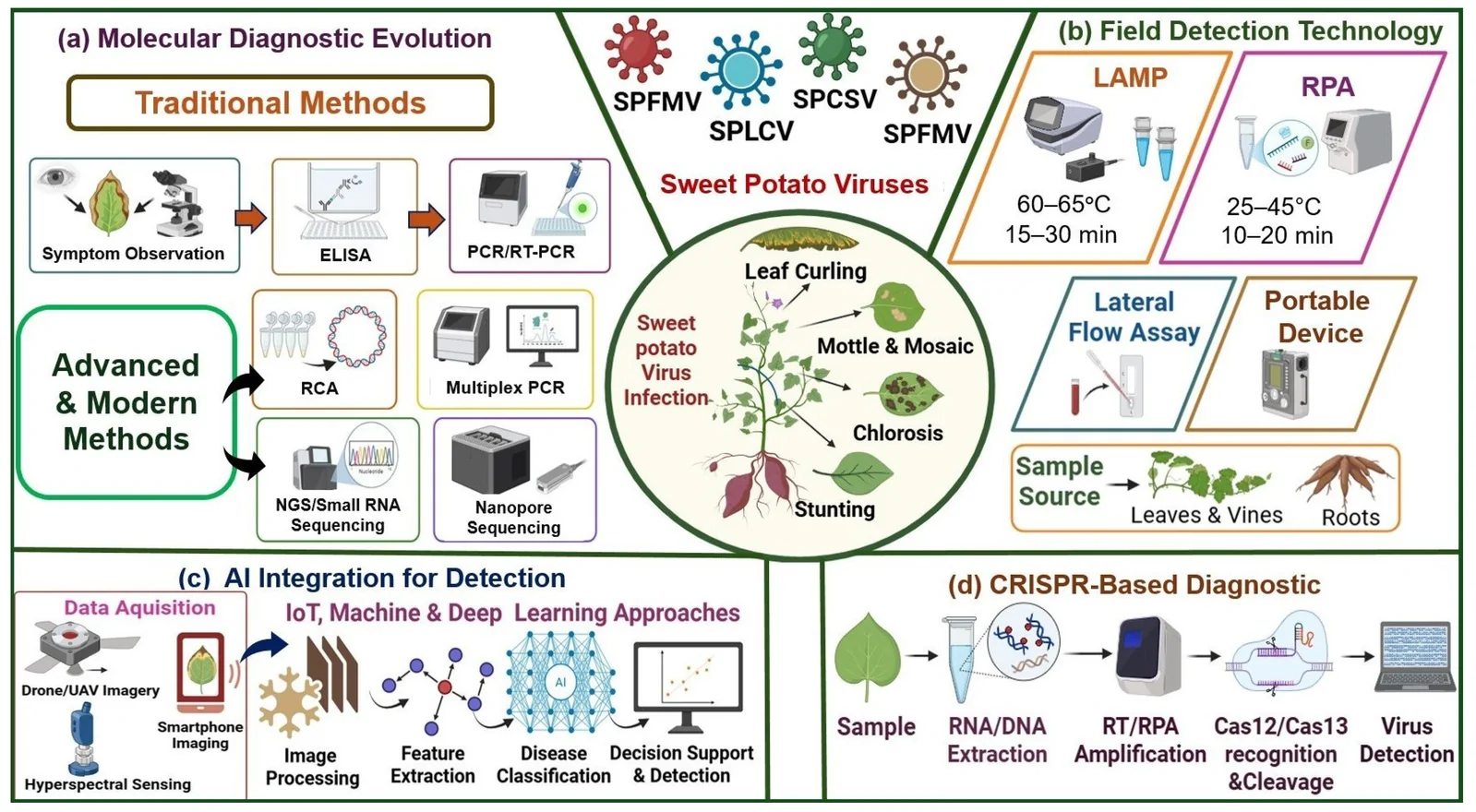
Schematic overview of molecular diagnostics strategies for sweet potato virus detection. (**a**) Traditional symptom-based observation, ELISA, and PCR/RT-PCR are shown alongside advanced methods including RCA, multiplex PCR, NGS/small RNA sequencing, and nanopore sequencing. (**b**) Field-deployable technologies such as LAMP, RPA, lateral flow assays, and portable devices are also shown. (**c**) AI-assisted image analysis is included for rapid visual detection. (**d**) CRISPR-based diagnostic workflows are highlighted for sensitive and specific virus detection. Created in BioRender. Smith, J. (2025). BioRender.com/c248457.

More recently, small RNA sequencing and genome-based methods have enabled simultaneous detection of multiple viruses and improved identification of mixed infections [19]. Recent studies have developed advanced diagnostic methods such as CRISPR (clustered regularly interspaced short palindromic repeats)-based detection, microarrays, and sequencing tools, which provide higher accuracy and can potentially be employed in the field [20,21]. These advances are important for sweet potato because they are often affected by multiple virus infections, making single-virus tests less reliable [19]. In addition, molecular techniques are integrated with imaging and machine learning to enable fast field screening before lab confirmation [18]. Figure 1 summarizes molecular diagnostics strategies for sweet potato virus detection.

This review provides a comprehensive and evidence-based overview of these recent advances in sweet potato virus detection, covering the progression from early PCR techniques to advanced CRISPR-based technologies. It highlights the advanced application of conventional, molecular, and emerging diagnostic methods for detecting sweet potato viral diseases, while discussing their advantages, limitations, future prospects, and current challenges.

## 2. Sweet Potato Viruses: A Brief Taxonomic Overview

Sweet potato is affected by more than 30 viruses from different families, making viral diseases a major production constraint worldwide [22,23]. Key pathogens belong to the family *Potyviridae*, including SPFMV, sweet potato virus G (SPVG), sweet potato virus C (SPVC), sweet potato virus 2 (SPV2), and sweet potato latent virus (SPLV) [23,24]. Disease complexes involving potyviruses and SPCSV are the most economically important. Mixed infections are generally more severe than single infections, reducing plant vigor and storage root yield.

Typical symptoms include chlorosis, mottling, vein clearing, leaf curling, deformation, and stunting. However, many viruses cause mild or latent symptoms, complicating field diagnosis. In mixed infections, disease severity increases significantly [15,24,25,26]. Due to variable symptom expression, molecular diagnostics are essential for accurate detection and differentiation. Table 1 summarizes major sweet potato viruses, their classification, transmission, symptoms, and economic importance.

## 3. Symptoms, Phenotypes and AI-Based Detection Methods

Before molecular techniques, virus detection relied on symptom observation, indicator plant bioassays, vector transmission assays, mechanical transmission tests, electron microscopy, and serological approaches, particularly ELISA. These methods established the foundation of virus diagnostics but have limitations in sensitivity, specificity, and scalability.

### 3.1. Symptom-Based Diagnosis

One of the first techniques for identifying sweet potato diseases was visual symptom assessment, which involved monitoring symptoms like chlorosis, mosaic, leaf curling, vein clearing, deformation, and stunting in the field [43]. This approach is simple and cost-effective but unreliable because many infections are mild or asymptomatic and easily confused with abiotic stresses. Symptom expression varies with virus type, cultivar, growth stage, and environmental conditions, and accuracy depends on observer expertise [7,8]. Studies confirm that mixed infections cause severe symptoms, whereas single infections may remain undetected, highlighting the need for molecular confirmation [44].

### 3.2. Biological Indexing

Biological indexing involves grafting infected tissue onto indicator plants such as *Ipomoea setosa* or *Ipomoea nil*, which express clear symptoms. It is more reliable than visual diagnosis but is time-consuming, labor-intensive, and cannot precisely identify virus species [45,46]. It remains valuable for detecting latent and phloem-restricted viruses like SPCSV. Studies demonstrate its effectiveness in identifying hidden infections and evaluating resistance, despite requiring several weeks and controlled conditions [47].

### 3.3. Electron Microscopy (EM)

Transmission electron microscopy (TEM) enables visualization of virus particles and cellular changes. Techniques such as negative staining and ultrathin sectioning provide structural and cytopathological insights, while immuno-negative staining improves specificity [48]. However, EM requires specialized equipment, has low sensitivity for low-titer infections, and cannot distinguish closely related strains without additional methods. It is mainly used for virus characterization rather than routine diagnostics [45].

### 3.4. Artificial Intelligence (AI) for Sweet Potato Virus Detection

AI has emerged as a transformative technology in sweet potato virus detection, enabling rapid, accurate, and non-destructive diagnostic solutions through advanced computational algorithms, machine learning, and deep learning approaches [8]. Machine learning techniques such as Support Vector Machines (SVM) classify infected and healthy plants based on image and spectral data, while deep learning frameworks, particularly Convolutional Neural Networks (CNNs), identify SPVD-infected leaves from field-captured images with high accuracy through automated feature extraction and lesion segmentation [49]. Ding et al. (2024) reported that their deep learning model achieved over 90% accuracy in identifying SPVD-infected leaves from field images, successfully segmenting virus lesions from healthy foliage with minimal false positives [49]. Zeng et al. demonstrated the feasibility of detecting SPVD from high-resolution aerial and ground-level imagery using deep learning frameworks, achieving detection accuracy of 89% and enabling large-scale field surveillance without laboratory infrastructure across multiple sweet potato growing regions [8]. Yang et al. (2026) developed novel 3D laser imaging combined with deep learning for nondestructive detection of SPLCV, capturing three-dimensional structural information about plant morphology and identifying virus-induced architectural changes such as leaf curling and stunting with detection sensitivity comparable to RT-PCR but without requiring sample extraction [18]. Zeng et al. (2025) created SPVD-DETR, a real-time end-to-end object detector based on transformer architecture for detecting SPVD from unmanned aerial vehicle ortho imagery, processing images at 30 frames per second and enabling aerial surveillance of virus-infected fields covering up to 50 hectares in a single flight [50]. These AI approaches provide early detection before visible symptoms, non-destructive analysis, rapid results within seconds, field-deployable smartphone applications, and over 50% cost reduction compared to traditional methods, making them essential for sweet potato seed certification, germplasm exchange, and precision agriculture programs [8]. Although AI-based approaches show great potential for sweet potato virus detection, their application is limited by the availability of high-quality training datasets, variability in field conditions, differences among cultivars, reduced accuracy during early stages of infection, and the inability of symptom-based classification to replace confirmatory molecular diagnostic methods [49]. On the other hand, AI-based visual detection tools can perform well when pathogens produce distinct and characteristic symptoms. However, their ability to distinguish closely related viruses may be limited when different pathogens produce overlapping symptoms. This is particularly relevant to sweepoviruses, a group of sweet potato-infecting begomoviruses comprising 14 currently ICTV-recognized species (International Committee for the Taxonomy of Viruses). Because sweepovirus infections can produce similar symptoms such as leaf curling, yellowing, vein yellowing, and stunting, visual AI models may have difficulty differentiating individual viral species without molecular confirmation [51,52].

## 4. Peptide-Based Methods

Among various peptide-based methods, ELISA is the most common and widely used technique. ELISA is a solid-phase immunoassay where a specific antibody binds the target antigen, and detection is achieved through an enzyme-catalyzed signal after the substrate is added. In virus detection, this assay is generally carried out in a sandwich antigen-capture format [51]. ELISA is commonly used for detecting viral antigens, offering cost-effective and large-scale screening [44,53]. It detects major sweet potato viruses and is suitable for surveys and certification programs. However, its sensitivity is limited for low-titer infections, and antibody availability restricts its application.

### 4.1. Combined ELISA Approaches in Molecular Studies

Recent studies have integrated ELISA with molecular and transcriptomic analyses. For example, Zhang et al. (2025) investigated transcriptomic and small RNA responses in sweet potato coinfected with SPCSV and SPFMV during storage root sprouting, using ELISA to confirm virus presence and higher virus titers in coinfected plants [54]. The study demonstrated that coinfection caused greater changes in gene expression and small RNA profiles than single infections, highlighting viral synergy and ELISA’s role in validating infection status in molecular studies [54]. David et al. (2023) used ELISA to detect and quantify SPCSV and SPVC in early-stage sweet potato seedlings for SPVD phenotyping, finding that co-infection caused more severe symptoms than single infections and that ELISA absorbance values correlated with symptom severity across genotypes, demonstrating that ELISA-based phenotyping is effective for early virus detection and selection of SPVD-resistant lines in breeding programs [22].

### 4.2. Double Antibody Sandwich ELISA (DAS-ELISA)

DAS-ELISA is a double-antibody sandwich immunoassay in which a capture antibody first binds the target antigen, followed by a second enzyme-linked detection antibody, producing a measurable colorimetric signal after substrate addition [55]. In a study DAS-ELISA was used to detect SPFMV, SPMMV, SPCSV, SPV2, and CMV revealed the presence of SPFMV and SPCSV in sweet potato plant that displayed typical virus-like symptoms. The study also demonstrated that DAS–ELISA was able to detect SPFMV and SPCSV in severely symptomatic plants without the need for prior grafting [56]. Kreuze et al. (2023) developed a high-throughput phenotyping and characterization system for resistance and tolerance to virus infection in sweetpotato, utilizing small RNA sequencing and assembly (sRSA) combined with DAS-ELISA-based validation for virus detection and quantification [12]. This approach enabled rapid screening of large breeding populations and identified genotypes with high resistance or high tolerance to SPVD [12]. Similarly, Srivastava et al. (2025) used DAS-ELISA for detecting sugarcane yellow leaf virus in symptomatic and asymptomatic sugarcane plants [57]. All 19 symptomatic samples tested positive by DAS-ELISA, confirmed by RT-PCR, demonstrating DAS-ELISA’s reliability for large-scale planting material certification [57]. In another study, DAS-ELISA with polyclonal antibodies against citrus tristeza virus (CTV) recombinant coat protein for early detection of CTV in Nepalese citrus, enabling rapid screening of mild, decline, seedling yellow, and stem pitting isolates, with RT-PCR validation improving diagnostic accuracy at low virus titers [58].

### 4.3. Nitrocellulose Membrane ELISA (NCM-ELISA)

NCM-ELISA employs a nitrocellulose membrane as the solid support, where viral antigens present in plant sap are immobilized. Specific antibodies then bind to these antigens, and an enzyme-substrate reaction produces a visible color signal, enabling the detection of plant viruses [59]. NCM-ELISA has been widely used in field surveys across Africa and Asia to detect SPFMV, SPCSV, SPVG, and related viruses [44,45]. Researchers have also combined NCM-ELISA with tissue-blot immunoassays to detect latent viruses in sweet potato meristem-derived plantlets, achieving higher detection rates than ELISA alone. Huang et al. (2023) used grafting-based bioassays combined with NCM-ELISA and qRT-PCR to evaluate SPVD resistance in 86 sweet potato varieties, identifying 17% of varieties as infected and confirming 83% as virus-free, with clear variation in resistance levels among genotypes, demonstrating that combining grafting with ELISA is an effective and sensitive approach for detecting latent infections and identifying SPVD-resistant and susceptible varieties [47]. However, current commercial NCM-ELISA kits detect only 10 of the nearly 30 known sweet potato viruses due to limited antisera availability, likely because many newly identified viruses have not been fully characterized. As a result, while major viruses such as SPFMV and SPCSV can be detected, other important viruses may be missed during diagnosis [45].

### 4.4. TAS ELISA, Dot-ELISA, and Advanced Variants

Another popular approach of ELSIA is Triple-Antibody Sandwich ELISA (TAS-ELISA) that uses three antibodies to catch and detect viral antigens, improving sensitivity and specificity versus DAS-ELISA. It successfully distinguishes multiple sweet potato viruses (SPCSV, SPFMV, SPVC) with detection in crude extracts demonstrating exceptional sensitivity for high-throughput screening in breeding programs and quarantine testing and is particularly valuable for detecting co-occurring viruses in mixed infections common in sweet potato [60]. Similarly, Dot-ELISA and Flower-ELISA have been developed as simplified versions requiring less equipment and suitable for field laboratories. For example, He et al. (2021) developed TAS-ELISA, dot-ELISA, and tissue-print-ELISA using monoclonal antibodies for detecting *Dickeya dadantii*, the causal agent of sweet potato stem and root rot [60]. TAS-ELISA and dot-ELISA detected *D. dadantii* in sweet potato crude extracts diluted up to 1:3840 and 1:1920 (wt/vol), respectively, and both were more sensitive than conventional PCR [60]. These techniques are also used in other crops viral detection such as Guo et al. (2023) developed monoclonal antibody-based Dot-ELISA for plum pox virus detection in apricot trees, achieving 10 ng/mL detection—significantly more sensitive than DAS-ELISA [61]. Dot-ELISA required less samples, was completed in 2 h, and was combined with colloidal gold immunochromatographic strips for naked-eye field detection [61]. Zhang et al. (2023) developed Dot-ELISA and gold nanoparticle strips for maize chlorotic mottle virus detection, achieving 0.5 ng/mL detection (100× more sensitive than conventional ELISA) [62]. The gold nanoparticle strip enabled naked-eye detection within 15 min without laboratory equipment [62]. Zhao et al. (2023) developed monoclonal antibody-based Dot-ELISA and colloidal gold strips for tomato brown rugose fruit virus, achieving 10 ng/mL detection and naked-eye visualization within 10 min [63]. Chen et al. (2020) compared IC-RT-PCR, Dot-ELISA, and Indirect-ELISA for sorghum mosaic virus detection [64]. IC-RT-PCR was most sensitive, followed by Dot-ELISA, with Indirect-ELISA least sensitive. Dot-ELISA provided the best balance of sensitivity, speed, and cost for routine field screening [64].

### 4.5. Advanced ELISA-Derived Technologies

Emerging ELISA-derived technologies exhibit exceptional sensitivity, reproducibility, and multi-target detection capabilities. Digital ELISA is a single-molecule immunoassay that isolates individual target molecules into separate reaction compartments, allowing highly sensitive quantification of viral proteins through digital counting instead of measuring an average bulk signal [65]. Digital ELISA enables single-molecule sensitivity, achieving detection limits 100–1000× lower than conventional ELISA by partitioning samples into thousands of microwells with antibody-coated magnetic beads for digital counting. While sweet potato virus applications are still emerging, digital ELISA has detected ultra-low virus titers in other crops’ symptomless or early-stage infections and could identify latent infections in sweet potato planting material that conventional ELISA misses, enabling virus-free seed systems [19,65]. Paper-based ELISA uses microfluidic paper-based analytical devices (μPADs) with patterned hydrophobic barriers directing fluid flow through antibody-coated detection zones [66]. The method achieved detection limits comparable to traditional ELISA using only capillary action, eliminating pumps and instrumentation. Paper-based ELISA is inexpensive, portable, room-temperature stable, and enables visual colorimetric detection with the naked eye, making it ideal for on-site sweet potato virus testing by farmers and extension workers in remote areas. Plasmonic ELISA uses gold nanoparticles and localized surface plasmon resonance (LSPR) for naked-eye detection at pico- to femtomolar concentrations (100–1000× more sensitive than conventional ELISA) [67]. Color changes from nanoparticle aggregation enable detection without instrumentation, and this technology can detect very low sweet potato virus titers in early infection stages, which is ideal for field use in resource-limited settings. Despite these advances, ELISA remains constrained by antibody availability and the need for laboratory infrastructure. A major limitation of ELISA-based molecular detection is the difficulty of distinguishing specific target-binding events from non-specific background signals [68]. ELISA can detect target antigens at low concentrations, but sensitivity varies substantially with assay format, antibody pair selection, and sample condition, so performance cannot be generalized across all ELISA platforms. Integrated detection workflows can enhance the utility of ELISA by combining it with molecular assays, allowing ELISA to function as an initial high-throughput screening method [69]. ELISA specificity can be affected by antibody cross-reactivity, sample matrix effects, and non-specific binding, which may cause false-positive results and reduce diagnostic accuracy, especially when similar antigens are present [70]. Future research should prioritize developing aptamer-based ELISA, field-deployable paper-based and plasmonic ELISA formats, and integrated ELISA-molecular platforms specifically validated for sweet potato virus detection in realistic field conditions.

## 5. Molecular Laboratory Methods

### 5.1. Single RT-PCR and PCR Assays

PCR amplifies DNA by repeated cycles of denaturation, annealing, and extension; RT-PCR first converts RNA into cDNA and then amplifies it; single RT-PCR performs reverse transcription and PCR in one tube for faster, less contaminated detection [71]. Single RT-PCR successfully detects SPFMV, SPCSV, SPVG, IVMV, and SPLCV directly from infected plants through RNA extraction, reverse transcription to cDNA, and PCR amplification with virus-specific primers that amplify nucleic acids at nanogram levels [54,72,73]. RT-PCR assays targeting *Potyvirus* conserved viral genes, including coat protein (CP), NIb, HC-Pro, and Rep genes, allow reliable amplification and are widely used for field identification [15,25,54]. PCR is particularly valuable for sweet potato, where high polysaccharide and starch content inhibits conventional extraction; simplified protocols minimize matrix interference while preserving nucleic acid quality. PCR sensitivity far exceeds serology, enabling detection at very low concentrations with primer-defined specificity [54,73].

Recent molecular surveys and detection method developments have considerably enhanced understanding of sweet potato virus diversity, transmission dynamics, and diagnostic capabilities. Small RNA deep sequencing combined with PCR/RT-PCR in southern China identified 12 virus species, with SPFMV (89.5%), SPCSV (52.6%), and SPVG (36.8%) as predominant pathogens, while Beijing surveys detected 10 virus species including SPFMV, SPCSV, and SPLCV, demonstrating geographic variation in virus prevalence [74,75]. Large-scale RT-PCR surveys across Korean fields (2011–2014) revealed SPFMV infection rates of 87% and SPVC of 85% among 154 sampled plants, with virus incidence increasing over the study period [29,76]. In South Africa’s KwaZulu-Natal province, RT-PCR confirmed SPFMV, SPVG, SPCSV, and SPMMV as most prevalent viruses following NCM-ELISA screening, with multiple virus infections common in commercial cultivars [45]. Similarly, Hungarian surveys detected and subsequently eliminated four major viruses (SPFMV, SPCSV, SPLCV, and SPVMV) through thermotherapy and meristem culture, achieving virus-free planting material [25]. Advanced detection methods include one-step RT-qPCR assays achieving detection limits of 10 pg/μL RNA for simultaneous identification of multiple viruses [77] and dual RT-RPA assays enabling field-deployable detection of SPFMV and SPCSV within 25 min at 39 °C, with sensitivity comparable to RT-PCR but without thermal cycling requirements [13]. Immunocapture PCR (IC-PCR) combines ELISA’s antigen capture with PCR amplification, allowing detection of viruses at very low titers while providing both serological specificity and molecular sensitivity, making it particularly valuable for detecting phloem-restricted viruses like SPCSV that are often missed by ELISA alone [78]. These molecular approaches provide detection sensitivity far exceeding ELISA detection limits and enabling early diagnosis before symptom expression. Tapily et al. (2025) used RT-PCR, sequencing, and ELISA to identify sweet potato viruses in Côte d’Ivoire, including SPCSV and SPFMV, revealing high genetic diversity among SPFMV isolates and demonstrating that molecular methods are important for detecting viral variation and novel strains, while ELISA remains useful for large-scale screening when suitable antibodies are available [6]. This genera-targeted approach extends the diagnostic utility of a single RT-PCR assay beyond species-specific primer sets.

### 5.2. Multiplex RT-PCR

Multiplex PCR is a molecular technique that uses target-specific primers to simultaneously detect multiple pathogens in a single reaction, with amplicons of different lengths designed to minimize cross-reactivity [71]. Multiplex RT-PCR assays have been successfully developed for detecting up to eight sweet potato viruses simultaneously, including SPFMV, SPCSV, SPVG, SPLCV, SPVMV, IVMV, and SPMMV, with detection limits at nanogram levels comparable to single-plex RT-PCR while reducing processing time by 60–70% [29,79]. TaqMan real-time multiplex RT-PCR assays targeting three *Potyviridae* viruses in sweet potato demonstrated enhanced sensitivity with detection limits of 10 pg/μL RNA and enabled precise discrimination between closely related potyviruses (SPFMV, SPCSV, and SPVMV) through distinct fluorescent probes [31]. The integration of magnetic nanobeads with multiplex RT-PCR further improved detection sensitivity by 10-fold and reduced assay time to under 2 h for simultaneous detection of multiple sweet potato viruses [80]. Field-deployable portable PCR systems have been developed for on-site sweet potato virus detection in Africa, achieving sensitivity comparable to laboratory-based RT-PCR while eliminating the need for centralized laboratory infrastructure and enabling rapid diagnosis within 3 h in remote production areas [81]. Recent advances include multiplex RT-qPCR assays detecting viruses from both whitefly vectors and plant samples simultaneously, directly linking vector transmission dynamics with plant infection status and enabling early detection before symptom expression [82], and riboprobe development for rapid field detection of seven SPVD-associated virus species including novel strains in Costa Rica [21]. Virus elimination programs using multiplex PCR-based screening have successfully achieved 100% virus-free planting material in Ethiopia through thermotherapy at 37 °C for 4–6 weeks combined with meristem culture, detecting and eliminating SPFMV, SPCSV, and SPLCV from high-yielding varieties with 95% recovery rates [56]. Similar multiplex approaches have been adapted for sunflower (5 viruses), cucurbits (4 viruses from whiteflies), sugarcane (5 viruses), tomato (6 RNA viruses), and lilies (4 viruses), demonstrating the broad applicability of this diagnostic strategy across crop systems [83,84,85,86]. These multiplex approaches, targeting CP, NIb, HC-Pro (potyviruses), and Rep (begomoviruses) genes, extend diagnostic utility beyond species-specific primer sets and provide comprehensive virus profiling essential for epidemiological surveys, germplasm certification, virus indexing programs, and crop protection strategies. 454-pyrosequencing combined with multiplex one-step RT-PCR has further characterized sweet potato virus C and SPFMV isolates from Argentina, revealing genetic diversity and enabling development of region-specific detection assays [87].

### 5.3. Real-Time Quantitative RT-PCR (qRT-PCR)

Quantitative real-time PCR (qPCR) detects and measures DNA amplification in real time using fluorescent dyes such as SYBR Green or probes like TaqMan. The fluorescence intensity increases proportionally with the amount of amplified DNA, allowing accurate quantitative analysis. Recent advances in RT-qPCR normalization and detection methods have significantly improved the accuracy and reliability of sweet potato virus gene expression studies. Critical work on reference gene validation identified stable internal controls for RT-qPCR normalization across different sweet potato tissues, with actin, ubiquitin, and EF1α demonstrating highest stability in leaves, roots, and storage roots, while virus-infected plants required tissue-specific reference genes (TUB and SAND) to avoid normalization artifacts from viral infection-induced transcriptional changes [88,89]. Triplex TaqMan qPCR assays have been established for simultaneous detection of multiple viruses with detection limits of 10 copies/reaction, demonstrating 100-fold greater sensitivity than conventional RT-PCR [90]. Similarly, Real-time reverse transcription recombinase polymerase amplification (RT-RPA) assays for cassava brown streak viruses achieved detection within 25 min at 39 °C without thermal cycling, providing a field-deployable alternative with sensitivity comparable to RT-Qpcr which can also be applied for sweet potato virus detection [91]. Another combined approach is ELISA-qRT-PCR, where ELISA is used for initial screening and qRT-PCR for confirmation, improving diagnostic accuracy. For example, Buko et al. (2024) conducted virus detection and elimination of SPFMV in high-yielding sweet potato varieties from Ethiopia using ELISA (specifically DAS-ELISA and NCM-ELISA) for initial virus screening, followed by RT-PCR confirmation [56]. The study found that ELISA was effective for screening large numbers of samples, while RT-PCR provided higher sensitivity for confirming virus elimination, particularly for detecting low-titer or latent infections [56]. qRT-PCR has also been widely used to validate transcriptomic datasets generated from virus-infected sweet potato plants. For example, expression analysis of 26 differentially expressed genes identified by RNA-seq in plants infected with SPFMV, SPVG, and SPV2 confirmed the sequencing results, demonstrating the accuracy and reliability of qRT-PCR for quantitative gene expression analysis during sweet potato-virus interactions [26,92]. The identification of symptomless badnaviruses in sweet potato has underscored the importance of highly sensitive qRT-PCR-based diagnostics for accurate virus detection and profiling [41]. Recent developments, including the validation of suitable reference genes and the establishment of multiplex triplex TaqMan qRT-PCR assays, have enhanced the sensitivity, specificity, and quantitative accuracy of virus detection. These advances have strengthened the application of qRT-PCR in virus surveillance, epidemiological studies, and the certification of virus-free planting materials [41].

### 5.4. Digital Droplet PCR (ddPCR)

Droplet digital PCR (ddPCR) is an advanced molecular assay that allows highly accurate detection and absolute quantification of specific DNA or RNA targets. Like RT-qPCR, ddPCR uses target-specific primers and fluorescent probes. However, unlike RT-qPCR, the reaction is partitioned into thousands of droplets for parallel amplification, with positive and negative droplets counted to achieve absolute quantification of target DNA or RNA using Poisson statistics [93]. Although this highly sensitive and versatile technology has been widely applied for mutation detection and copy number analysis in the human genome, only a limited number of studies have reported the use of RT-ddPCR for plant virus detection [94]. ddPCR has been applied in plant virus diagnostics for highly sensitive and absolute quantification, including RT-ddPCR assays developed for plum pox virus and cucumber green mottle mosaic virus [94,95].

While ddPCR is increasingly applied in plant virology, published ddPCR data for sweet potato viruses appears to be scarce or unavailable. Sweet potato virus detection has instead been reported more frequently using qPCR and multiplex RT-PCR platforms for major viruses. Conventional PCR is effective for detecting known viral targets, but its specificity depends on careful primer design. Poor primer matching or the presence of similar sequences can result in non-specific amplification or false-negative results [96]. Although RT-PCR improves detection of RNA viruses by adding a reverse transcription step, yet its diagnostic reliability still depends on RNA quality, reverse-transcription efficiency, and the absence of inhibitors in the sample matrix [97]. Both conventional PCR and RT-PCR can produce false-positive results due to amplicon contamination; therefore, proper laboratory practices, including physical separation of workflows and the use of negative controls, are necessary to ensure reliable results [98]. Mutations in primer-binding sites can also reduce sensitivity and cause false negative results. The PCR based method is best considered a sensitive confirmatory method rather than a broadly applicable field diagnostic approach, especially in cases involving low viral concentrations, sample inhibitors, or mixed infections [98].

## 6. Development of Advanced Biosensors

### 6.1. Loop-Mediated Isothermal Amplification (LAMP)

LAMP employs a DNA polymerase with strand displacement activity, along with four to six specially designed primers that recognize six to eight regions of the target DNA, to amplify target nucleic acids at a constant temperature (typically 60–65 °C), eliminating the need for a thermocycler [99]. It rapidly amplifies DNA through stem-loop structures, producing large amounts of DNA within 15–60 min, making it suitable for on-site detection providing a fast and simple diagnostic tool for pathogen detection. LAMP also produces large quantities of amplification products, which can be detected visually as turbidity from magnesium pyrophosphate precipitation, as fluorescence with intercalating dyes (e.g., SYBR Green), or through colorimetric indicators (such as calcein and naphthol blue), or by lateral flow devices [99,100,101].

LAMP has emerged as a rapid, sensitive, and field-friendly method for sweet potato virus detection, offering a practical alternative to conventional PCR in seed health testing and on-site diagnostics. RT-LAMP assays have been successfully developed for detecting major sweet potato viruses including SPFMV, SPCSV, and other sweet potato-infecting viruses, with detection limits comparable to RT-PCR but without requiring thermal cycling equipment [102,103]. Ultra-portable pocket-sized devices have been evaluated for running LAMP assays in the field, demonstrating that sweet potato viruses can be detected rapidly using battery-powered, handheld real-time systems suitable for remote production areas in Africa and other developing regions [104]. LAMP assays have been integrated into sweet potato seed quality assurance programs, enabling rapid verification of virus-free planting material and supporting certification schemes that are critical for maintaining yield potential and preventing virus spread through vegetative propagation [105]. Similar LAMP platforms have been validated for detecting Mycobacterium in bovine feces using portable real-time systems, demonstrating the broader applicability of this technology for point-of-care diagnostics across agricultural and veterinary settings [106]. RT-LAMP has also been developed for sweet potato latent virus detection in lotus plants, achieving rapid identification within 45 min with high specificity and sensitivity [107]. Visual LAMP detection methods have been established for potato virus Y, providing colorimetric readouts that eliminate the need for fluorescence detection equipment [108]. Microfluidic integration through roll-to-roll fabrication of PDMS–paper devices has enabled nucleic acid amplification in compact, disposable formats suitable for field deployment [109]. LAMP assays have been validated for bitter gourd viruses (coccinia mosaic virudhunagar virus) and cassava component detection in sweet potato noodles, demonstrating the versatility of this approach across diverse crop systems and food safety applications [110,111]. Field detection technologies such as portable LAMP and image-based approaches emerging as promising tools for rapid field screening, whereas RT-PCR and qPCR remain the established laboratory methods for confirmation and quantification. Rather than replacing these established methods, portable diagnostic tools complement them by enabling rapid testing in field settings while laboratory-based assays provide confirmatory support [69]. Overall, LAMP technology represents a critical advancement in sweet potato virus diagnostics, bridging the gap between laboratory-based molecular methods and practical field applications for early detection, seed certification, and disease management in sweet potato production systems worldwide. Despite these advantages, LAMP requires careful primer design targeting 6–8 specific regions of the viral genome to ensure specificity, and nonspecific amplification can occur if reaction conditions are not optimized, necessitating validation against known positive and negative controls [102,112]. Thus, the complexity of primer design, susceptibility to nonspecific amplification and false-positive results, and difficulties in multiplexing are some major limitations of LAMP [112].

### 6.2. Rolling Circle Amplification for DNA Virus Detection

Rolling circle amplification (RCA) is a technique used to amplify circular DNA molecules using random hexamer or short degenerate primers and the strand-displacing Phi29 DNA polymerase from *Bacillus subtilis* bacteriophage Phi29, generating long concatemeric DNA products composed of repeated copies of the circular template. Unlike conventional PCR, RCA specifically targets circular DNA and mimics natural DNA replication processes making it particularly suited for the detection of geminiviruses (begomoviruses/sweepoviruses) and other circular ssDNA viruses such as nanoviruses [100,113]. RCA has emerged as a powerful tool for detecting circular DNA viruses infecting sweet potato, particularly begomoviruses (e.g., sweet potato leaf curl virus) and badnaviruses, which possess circular or partially circular genomes. Recent studies have optimized RCA protocols for comprehensive detection and characterization of sweet potato badnaviruses, demonstrating that RCA efficiently amplifies full-length viral genomes from infected plant tissue, enabling downstream applications such as cloning, sequencing, and phylogenetic analysis [114]. RCA has been particularly valuable in virome analyses using HTS, where it facilitated the discovery of novel sweet potato viruses in Brazil, Europe, and Burkina Faso, including Sweet Potato Symptomless Virus 1 and unexplored pathogens previously undetected by conventional methods [42,115,116]. Regional surveys employing RCA-based approaches revealed novel isolates and genetic diversity of sweet potato begomoviruses in South Africa, China, and the United States, including natural recombinants between *Sweet potato leaf curl virus* and *Sweet potato leaf curl Georgia virus* [117,118,119]. However, RCA has notable limitations such as nonspecific amplification products are frequently generated, requiring additional methods such as restriction fragment length polymorphism (RFLP) analysis or sequencing to identify infecting viruses at the species or strain level; additionally, long incubation times (18–20 h) are required for amplification, limiting its utility for rapid field diagnostics [114]. Despite these drawbacks, RCA remains indispensable for comprehensive viral genome characterization, recombination analysis, and discovery of novel circular DNA viruses in sweet potato, complementing rapid detection methods like RT-PCR, RT-qPCR, and RT-RPA for integrated virus diagnostics [47,120].

### 6.3. Recombinase Polymerase Amplification (RPA)

Recombinase polymerase amplification (RPA) is an isothermal amplification technology of nucleic acid detection method that uses a recombinase, single-stranded DNA binding protein (SSBP), strand-displacing DNA polymerase, and 30–35 bp target-specific primers. The recombinase forms a complex with primers, which scans and invades double-stranded DNA at complementary sites; single-stranded DNA binding protein (SSBP) stabilizes the displaced strand, enabling exponential amplification without thermal cycling [121]. RPA operates at flexible temperatures (22–45 °C) and typically completes within 20 min. For RNA detection, reverse transcriptase is added directly to the reaction mix, making RPA a fast, isothermal alternative to PCR for both DNA and RNA viruses. RPA products are commonly detected by gel electrophoresis, real-time fluorescence, or lateral flow strips (LFS). RPA-LFS uses colloidal gold–labeled antibodies to visually detect amplification products as bands, offering a simple, instrument-free, and highly sensitive method suitable for rapid on-site pathogen and virus diagnosis [122,123].

**Figure 2:**
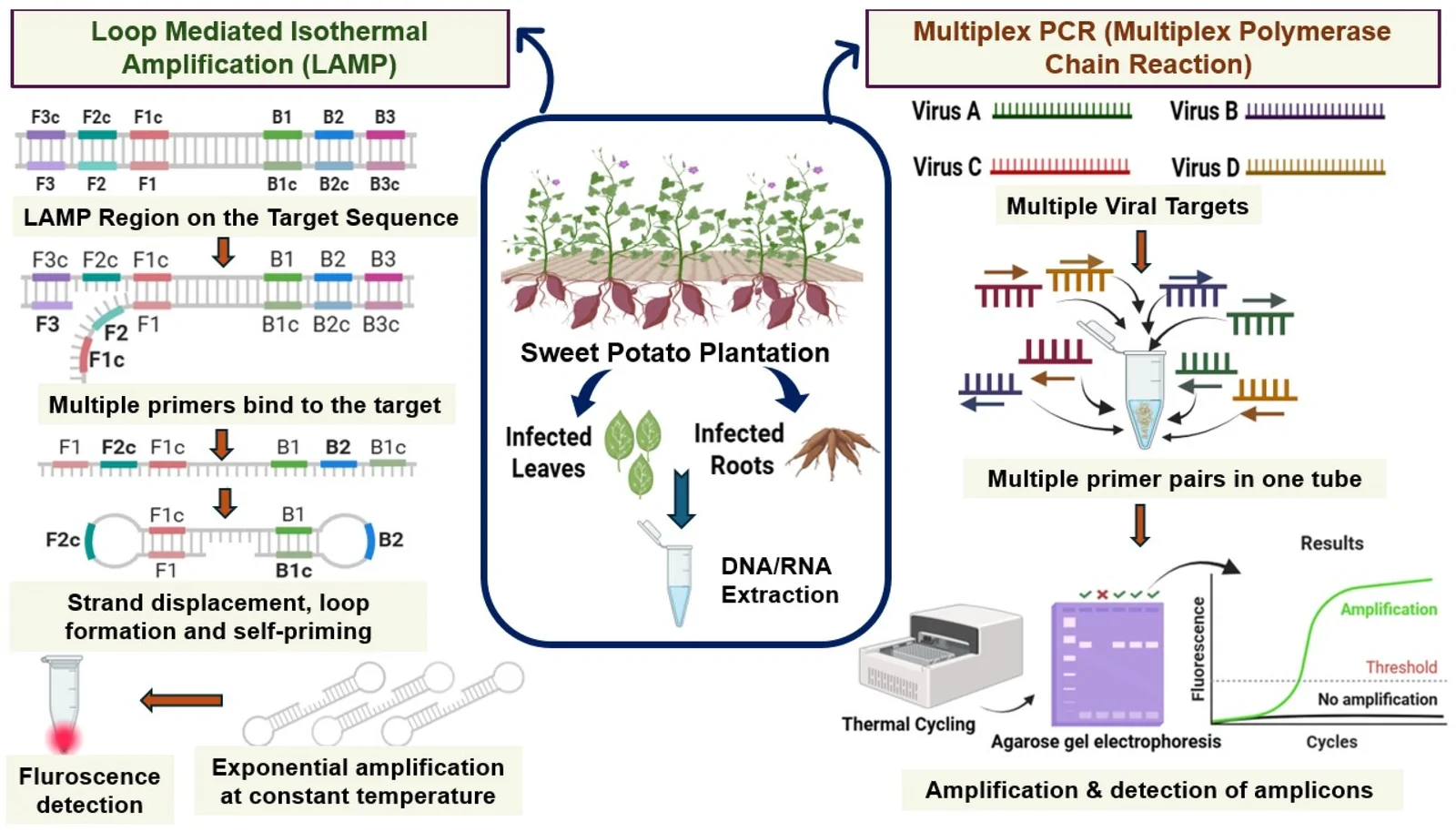
Principle of molecular diagnostic methods for sweet potato virus detection. LAMP is based on multiple primers, strand displacement, loop formation, and constant-temperature amplification with fluorescence detection. Multiplex PCR uses multiple primer pairs in one tube to amplify several viral targets simultaneously, followed by thermal cycling and amplicon detection. Created in part using BioRender.com (https://www.biorender.com/) and assembled and finalized by the authors using Microsoft PowerPoint. Created in BioRender. Smith, J. (2025). BioRender.com/c248457.

Recombinase Polymerase Amplification (RPA) offers significant advantages for sweet potato virus diagnostics, particularly in field-deployable applications. Dual RT-RPA assays have been successfully developed for simultaneous detection of SPFMV and SPCSV, achieving detection within 20 min at 39–42 °C without thermal cycling, with sensitivity comparable to RT-PCR but requiring minimal equipment [13]. Lateral flow dipstick-based RPA enables rapid sweepovirus detection with visual readouts, eliminating the need for fluorescence detection instruments and making it suitable for resource-limited settings [111]. RPA’s flexibility extends across plant viruses such as RT-RPA assays have been developed for potato virus Y detection in potato with high sensitivity, potato leafroll virus diagnosis, potato virus S detection using one-step RT-RPA, and cassava brown streak virus detection in real-time format [91,111,124]. It indicated that the technique can be easily adapted for other sweet potato viruses. These advantages make RPA particularly valuable for sweet potato seed quality assurance, early disease detection before symptom expression, and on-site surveillance in farmer fields where rapid decision-making is critical for preventing virus spread through vegetative propagation. Figure 2 illustrates the basic principle of LAMP and PCR.

Although these methods amplify target sequences under constant-temperature conditions, their reliability is influenced by primer selection and careful validation against similar genetic targets [125,126]. Isothermal amplification offers strong potential for field-based diagnostics because it eliminates the need for thermal cyclers and supports use in low-resource environments. However, this operational simplicity does not eliminate the need for rigorous specificity testing and contamination control [126,127]. Some isothermal assays may amplify off-target sequences or show variable performance across sample types, especially when sequence diversity or mixed infection is present [128]. Overall, isothermal amplification is well suited for rapid screening and decentralized testing, while RT-PCR, RT-qPCR, or sequencing remain important for confirmation and discrimination of closely related viruses [125].

### 6.4. CRISPR/Cas-Based Virus Detection

**Figure 3:**
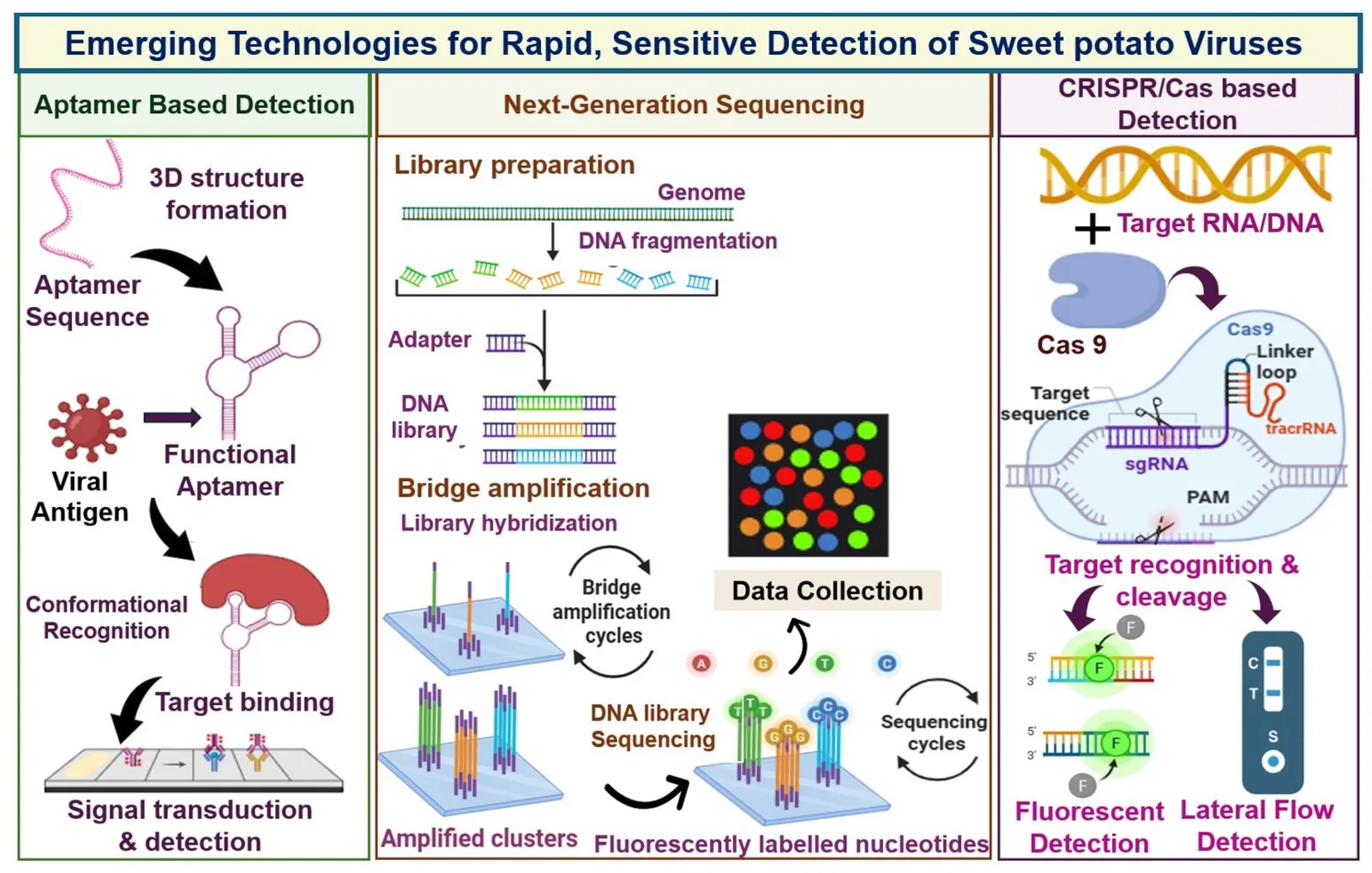
Emerging technologies for rapid, sensitive detection of sweet potato viruses. Aptamer-based detection relies on aptamer sequence folding, target antigen binding, conformational recognition, and signal transduction for virus detection. Next-generation sequencing involves DNA fragmentation, adapter ligation, library preparation, bridge amplification, sequencing, and data collection for comprehensive virus identification. CRISPR/Cas-based detection uses target RNA/DNA recognition by Cas9-sgRNA complexes followed by cleavage and readout by fluorescent or lateral flow detection. Created in BioRender. Smith, J. (2025). BioRender.com/c248457.

CRISPR/Cas-based virus detection in sweet potato leverages the adaptive immune system of bacteria and archaea, in which CRISPR-associated nucleases use guide RNAs to recognize and cleave specific nucleic acid targets [129]. Effector proteins such as Cas12a and Cas13a exhibit collateral (trans-cleavage) activity upon specific target binding; Cas12a primarily cleaves single-stranded DNA, whereas Cas13a targets single-stranded RNA following cis-cleavage [130]. These properties have enabled the development of powerful diagnostic platforms, including SHERLOCK (Cas13-based), DETECTR (Cas12-based), and HOLMES, which integrates Cas12a with isothermal amplification strategies such as LAMP or RPA for rapid and sensitive nucleic acid detection [131,132]. In typical workflows, viral nucleic acids are first amplified using RPA or RT-RPA, and in some formats further processed via T7 transcription before detection. The activated Cas enzymes then cleave labeled reporter molecules, generating a detectable fluorescent or visual signal [26,133]. These CRISPR-based assays have demonstrated high sensitivity and specificity, often surpassing conventional molecular diagnostics. Such systems have already been successfully adapted for plant virus detection, including apple-infecting RNA viruses and viroids [134] as well as begomoviruses such as tomato yellow leaf curl virus (TYLCV) and tomato leaf curl New Delhi virus (ToLCNDV) [135]. In sweet potato, however, direct application remains limited, although patent literature has described CRISPR-based detection approaches for sweet potato leaf curl virus (SPLCV) without amplification steps [7]. Furthermore, to the best of our knowledge, there are currently no peer-reviewed reports describing the application or evaluation of CRISPR-based diagnostics in combination with PCR or qPCR for the detection of sweet potato viruses. In addition, CRISPR-Cas13 has been explored for engineering resistance against sweet potato virus disease by targeting SPCSV-RNase3, thereby reducing viral suppressor activity and enhancing host resistance [136]. Despite these advances, several technical challenges remain, including the dependence on pre-amplification for low-abundance targets, the need for rigorous optimization of primers and crRNAs, and the lack of standardized, field-ready platforms. Addressing these limitations will be essential for translating CRISPR-based diagnostics into portable, reliable tools for sweet potato virus detection, particularly when compared with established methods such as RT-qPCR [137,138]. Figure 3 shows emerging technologies for sweet potatoes virus detection.

## 7. DNA-Sequencing-Based Methods and Metagenomics

### 7.1. HTS Platforms

HTS, also known as Next-generation sequencing (NGS) or RNA-seq technology, includes whole-genome, metagenomic, and targeted sequencing approaches. It plays an important role in detection of sweet potato disease by identifying unknown pathogens and accurately distinguishing mixed infections [139]. HTS involves sequencing the total nucleic acids extracted from a plant sample, followed by bioinformatic analysis to identify both known and novel viruses and viroids. The typical workflow includes sample preparation, nucleic acid extraction, optional viral nucleic acid enrichment, sequencing, and subsequent data analysis and validation [139,140]. The advent of HTS platforms such as Illumina, 454 pyrosequencing, Ion Torrent, Oxford Nanopore, and others has transformed plant virology by enabling the comprehensive, unbiased characterization of viromes directly from plant tissues without prior cultivation or molecular cloning [140].

In sweet potato research, HTS studies in southern China and Beijing identified 12 and 10 virus species respectively, with SPFMV, SPCSV, and SPVG as predominant pathogens, revealing significant geographic variation in virus prevalence [74,75]. Virome analysis across three Brazilian regions using HTS detected diverse sweet potato viruses including begomoviruses, potyviruses, and badnaviruses, while HTS in Greece elucidated the sweet potato virome with complete genome assemblies [115,141]. In Korea, HTS enabled completion of Sweet potato chlorotic stunt virus genomes, confirming virus presence and characterizing genetic variation [142]. HTS has also confirmed multiple co-infections of begomoviruses and potyviruses in sweet potato varieties under field conditions, correlating infection complexity with yield reduction [117]. Recent HTS analyses in Uganda revealed factors driving sweet potato virus evolution through NGS and genetic analyses [143]. Another study Nakasu et al. combined HTS with ELISA to analyze the sweet potato virome in Brazil, identifying eight major viruses including SPCSV, SPFMV, SPVG, SPLCV, and CMV. The study showed that HTS enables comprehensive detection of known and novel viruses, while ELISA and qRT-PCR remain crucial for validation of HTS results [115]. These advances position HTS as a universal diagnostic platform for comprehensive virus detection, genome reconstruction, and epidemiological surveillance in both known and novel plant viruses. The major strength of HTS is its comprehensive detection capability, allowing the simultaneous identification of mixed infections and novel or previously uncharacterized viruses, although the biological interpretation of low-titer viral sequences may require careful validation and advanced bioinformatic analyses [144].

### 7.2. Nanopore Sequencing for Real-Time Diagnostics

Nanopore sequencing is a single-molecule technique that detects nucleic acids by measuring changes in ionic current as DNA or RNA passes through a nanopore under an applied voltage. Motor proteins such as helicases guide single-stranded nucleic acids through the pore, where different bases produce distinct current disruptions, enabling real-time base identification and sequence analysis [145,146]. Nanopores are classified as biological (protein- or peptide-based structures in lipid membranes) or solid-state (engineered materials such as silicon oxide or graphene) [147,148]. Developed by Oxford Nanopore Technologies (e.g., MinION, GridION, and PromethION), it enables rapid, real-time, long-read sequencing without the need for large sequencing facilities [149]. These sequencing technologies are widely used in genome analysis, disease diagnosis, and environmental monitoring.

In sweet potato, this approach has been used to characterize novel viral threats and unexplored pathogens from Burkina Faso, including detailed analysis of CRESS-DNA viruses across 98 symptomatic samples, supporting broader virome analysis and improved genome-level resolution of infection diversity [116]. The technology’s portability exemplified by the MinION device and speed, with results available within hours rather than days, make it especially useful for surveillance and diagnostic work in field or resource-limited settings where rapid decision-making is critical for preventing virus spread. Beyond sweet potato, Nanopore has been successfully applied to diagnose other plant diseases and detect plant pathogens in both plant and insect tissues, achieving detection sensitivity comparable to conventional methods while providing full-length sequence data [150,151]. It has also been used to characterize and genotype potato virus Y isolates in potato, demonstrating its utility for virus identification, strain discrimination, and outbreak monitoring with accuracy comparable to Illumina sequencing but at substantially lower cost and faster turnaround time [152,153]. Nanopore sequencing provides advantages in portability, speed, long-read capability, and cost-effectiveness. However, the technology is still in developmental stage with several challenges. While raw-read accuracy has improved to 99%, it still lags behind Illumina short-read sequencing (>99.9%) with more complex error profile and requires high-quality samples, including high molecular weight DNA and full-length RNA, skilled technicians for operation and maintenance. Despite these limitations, ongoing improvements in sequencing accuracy, throughput, algorithm optimization, multiscenario applications, and programmable computing continue to expand its potential applications [7].

### 7.3. Small RNA Deep Sequencing (SRDS)

RNA interference (RNAi) is a major antiviral defense mechanism in plants, where small RNAs specifically target and degrade viral transcripts to suppress virus infection and spread. Virus-infected plants accumulate virus-derived small interfering RNAs (vsiRNAs), which can be recovered through deep sequencing of the small RNA population (sRNAome). This approach enables sequence-independent detection of both RNA and DNA viruses and allows complete virome reconstruction, even in mixed infections [154]. The process generally involves small RNA isolation, cDNA library construction, and HTS for comprehensive virus identification [154]. SRDS has emerged as a powerful, sequence-independent approach for sweet potato virus detection and virome reconstruction. SRDS studies in southern China identified 12 virus species infecting sweet potato, while Beijing surveys detected 10 virus species [74,75]. In Barbados, sRSA revealed multiple begomoviruses, potyviruses, badnaviruses, and mastreviruses in the sweet potato leaf virome, demonstrating SRDS’s ability to detect diverse virus families simultaneously [155]. SRDS has also been applied to characterize transcriptome and small RNA changes during SPCSV and SPFMV co-infection during sweet potato storage root sprouting, providing insights into virus-host interactions and antiviral defense responses [54,156]. The technique’s efficiency extends beyond sweet potato for example SRDS detected a potential new virus in *Hevea brasiliensis* (rubber tree) using small RNA deep sequencing combined with PCR validation [157], reconstructed viromes in mixed infections of cultivated *Solanum* plants characterizing antiviral defense mechanisms [158] and identified viruses responsible for lettuce big vein disease in Spain [159]. SRDS recovers virus-derived small interfering RNAs (vsiRNAs) spanning entire RNA and DNA virus genomes without prior knowledge of which virus is present, enabling complete virome reconstruction in mixed infections and providing a universal detection platform for both known and novel plant viruses [160].

NGS and viral metagenomic approaches provide broad, unbiased detection capabilities and are powerful tools for pathogen discovery and surveillance. However, their diagnostic performance is influenced by factors such as sequencing depth, the completeness and accuracy of reference databases, and bioinformatic processing pipelines. Therefore, untargeted sequencing approaches should not be assumed to provide the same level of analytical specificity as targeted molecular assays designed for defined viral sequences [161].

## 8. Biosensing Technology

Biosensing technology is an emerging approach for sweet potato virus detection, providing rapid, sensitive, and portable diagnostic solutions suitable for field applications. It combines a biological recognition element (antibodies, DNA probes, enzymes, or aptamers) with a physicochemical transducer to convert biological interactions into measurable signals [162]. Electrochemical biosensors detect electrical changes such as current, voltage, or impedance during virus recognition. Optical biosensors measure variations in light absorption, fluorescence, or refractive index caused by biological interactions. DNA/RNA-based affinity biosensors identify viral nucleic acids through specific hybridization with immobilized probes. Nanomaterial-based biosensors employ nanoparticles, nanotubes, nanowires, or quantum dots to enhance sensitivity and detection efficiency. Phage-based and electronic nose (E-nose) biosensors utilize bacteriophages or volatile compound detection systems for rapid and on-site virus identification [162,163]. Biosensing technology has emerged as a transformative approach for sweet potato disease detection, offering rapid, sensitive, and non-destructive diagnostic solutions. Emerging biosensing technologies, including portable imaging systems, isothermal amplification methods, and CRISPR-based molecular detection, are transforming sweetpotato virus diagnostics. In particular, portable LAMP-based platforms enable rapid, on-site detection, reducing reliance on conventional laboratory-based PCR and ELISA workflows. This shift supports timely diagnosis during seed certification, disease surveillance, and early outbreak response, thereby improving disease management. However, these technologies are not intended to replace established diagnostic methods immediately. Instead, their broader implementation depends on rigorous field validation and performance benchmarking against standard laboratory assays, including PCR and RT-PCR, to ensure their reliability and diagnostic accuracy [7,22]. Recent studies have applied quartz crystal microbalance (QCM) biosensors for sweet potato disease detection, including identification of sweet potato black spot disease caused by *Ceratocystis fimbriata* using QCM array technology, and non-destructive detection of volatile compounds (trans-caryophyllene and linalool) in early-stage black spot disease using QCM gas sensors based on modified MOF composites [164,165,166]. While direct biosensing applications for sweet potato virus detection remain limited, the technique’s success in plant pathology demonstrates its potential for viral diagnostics. Beyond sweet potato, biosensing has been successfully applied to detect sorghum mosaic virus using rapid fluorescence switch-on biosensors, bean pod mottle virus using virus-specific nanocavities with high specificity, Biosensing approaches offer advantages including portability, results within minutes, no requirement for thermal cycling, and potential for field-deployable diagnostics in resource-limited settings, making them promising for sweet potato virus surveillance and seed certification programs [167]. Despite their potential, many biosensor platforms developed to date remain limited by high cost, complex designs, multiple processing steps, and the need for skilled operators, which restricts their suitability for rapid, field-based plant virus detection [168].

### Aptamer-Based Detection

Aptamer-based detection is a powerful diagnostic platform that uses single-stranded DNA or RNA molecules (aptamers) selected through SELEX (Systematic Evolution of Ligands by Exponential Enrichment) to bind to specific targets, including viruses, proteins, and nucleic acids, with high affinity and specificity [165,169]. Aptamers are synthetic recognition elements that fold into unique three-dimensional structures, offering high stability at room temperature, high sensitivity reaching picomolar to femtomolar concentrations, low immunogenicity, and easy manufacturing compared to traditional antibodies [169,170]. These aptamers can be integrated into multiple biosensor formats, including aptamer-based ELISA (ELASA), electrochemical biosensors, surface plasmon resonance (SPR) imaging, fluorescent and colorimetric biosensors, and lateral flow devices for point-of-care testing [169]. In plant pathogen detection, aptamer-based sensing has been successfully applied to detect apple stem pitting virus (ASPV), where DNA aptamers PSA-H and MT32 detected ASPV coat proteins with higher sensitivity than monoclonal antibodies, and MT32 detected a broader range of genetically distinct ASPV isolates [171]. Furthermore, aptamers have been developed for detecting several other plant pathogens, including soybean rust [172]. The technology has also been adapted for tomato yellow leaf curl virus, and tomato mottle virus demonstrating its versatility for plant virus detection [173]. For sweet potato virus detection, aptamer-based platforms can be adapted to detect SPFMV, SPCSV, SPVG, SPVC, and other latent viruses with high sensitivity. For example, Yildirim-Tirgil (2023) showed that aptamer-based ELISA provides 100-fold higher sensitivity and a detection limit of 0.05 ng/mL compared to conventional ELISA, making it effective for detecting low-titer and latent sweet potato viruses such as SPCSV [170]. Aptamer-based detection is therefore described as a suitable recognition platform that complements ELISA and molecular assays, especially where rapid adaptation to new viral variants is needed [174]. Thus, it offers portability, low cost, room-temperature stability, and field-deployability, making it ideal for on-site testing by farmers and extension workers without laboratory infrastructure, potentially revolutionizing sweet potato virus diagnostics by enabling rapid, sensitive, and cost-effective screening of planting material and breeding programs.

## 9. Comparative Assessment of Molecular Detection Platforms

Molecular detection of sweet potato viruses has advanced from symptom-based and serological methods to highly sensitive nucleic-acid techniques. ELISA remains useful for routine and low-cost screening, while PCR and qRT-PCR provide greater sensitivity and specificity for accurate diagnosis. Multiplex RT-PCR enables simultaneous detection of multiple viruses, which is important in mixed infections. Isothermal methods such as LAMP and RPA are rapid and field-deployable because they do not require thermal cyclers. Sequencing approaches, including small RNA and nanopore sequencing, offer comprehensive virus discovery and genome analysis but require higher cost and expertise. Biosensors, AI-based imaging, and CRISPR/Cas diagnostics provide rapid and portable detection options, although they are still emerging as complementary tools to established molecular methods. Each molecular detection platform presents a distinct balance of sensitivity, specificity, cost, and field deployability, and the appropriate methods tailored to specific diagnostic objectives are summarized in Table 2.

## 10. Integration of Molecular Detection in Virus Elimination Programs

High-temperature treatment can promote plant recovery from viral infections by reducing viral replication and limiting virus movement into newly developing meristematic tissues [184,185]. Meristem tip culture is commonly combined with thermotherapy to regenerate plant material with reduced or eliminated virus load, and the success of the procedure depends on subsequent diagnostic confirmation. The regenerated plants must then be screened to confirm successful elimination and to ensure that only virus-free materials are advanced for propagation.

Molecular detection methods play a central role in this verification step. In one study, PCR and qPCR identified seven viruses, including SPCSV, SPVG, SPVC, SPFMV, SPV2, SPLCV, and SPPV, in single or mixed infections, and elimination after heat treatment plus meristem tip culture was confirmed by PCR and qPCR in five of six sweet potato genotypes, with SPPV remaining in some cases [31]. For potyviruses and begomoviruses, sensitive PCR-based assays, including multiplex PCR, are useful for routine post-treatment indexing. Multiplex RT-PCR has also shown greater sensitivity than ELISA, particularly when combined with nested PCR, highlighting the advantage of molecular tools over serological screening [79,186,187]. NGS provides a broader and highly sensitive option for detecting known and unexpected viruses in elimination programs [188]. In addition, LAMP has been proposed as a practical quality control tool for virus-free planting material certification because it combines high sensitivity, affordability, and suitability for low-resource settings [105]. However, SPPV remains a major limitation in sweetpotato virus elimination programs because it can persist after heat treatment and may escape detection by conventional assays, making complete virus-free certification difficult [41].

## 11. Future Perspectives and Emerging Technologies

Future advances in sweet potato virus diagnostics are expected to move toward integrated, rapid, low-cost, and field-deployable platforms capable of simultaneously detecting multiple viruses with high sensitivity and specificity. Although conventional RT-PCR and qRT-PCR remain the gold standards for laboratory-based detection, emerging technologies such as CRISPR/Cas diagnostics, nanopore sequencing, biosensors, and AI-assisted imaging are likely to transform disease surveillance and management systems.

One of the most promising future directions is the development of CRISPR-Cas diagnostic systems, including SHERLOCK, DETECTR, and related Cas12/Cas13-based platforms. CRISPR/Cas12a- and Cas13-based platforms combined with isothermal amplification methods such as RPA and LAMP could enable ultrasensitive, rapid, and portable diagnostics suitable for on-site testing in resource-limited regions where sweet potato is predominantly cultivated.

On the other hand, ddPCR has not yet been widely applied for sweet potato virus diagnostics, its exceptional sensitivity and ability to provide absolute quantification without the need for standard curves offer considerable potential for future applications. ddPCR could facilitate the detection of latent or low-titer virus infections, improve the accuracy of clean seed and virus-free planting material certification programs, and enable precise quantification of viral loads in single and mixed infections. Furthermore, its high tolerance to PCR inhibitors and capacity to detect minor viral variants make ddPCR a promising tool for epidemiological surveillance, disease monitoring, and studies of virus-virus and virus-host interactions in sweet potato.

The integration of NGS, small RNA deep sequencing, and nanopore sequencing into routine surveillance programs will greatly enhance the identification of emerging viruses, recombinants, and mixed infections that are difficult to detect using targeted assays [115,140]. Portable nanopore platforms such as MinION have potential for real-time virus monitoring and quarantine applications because they provide long-read sequence data directly in the field. However, improvements are still needed in sequencing accuracy, bioinformatics pipelines, sample preparation protocols, and cost reduction before large-scale implementation becomes feasible in developing countries [7,116,148].

Another promising direction is the development of biosensor- and aptamer-based diagnostic systems. Electrochemical, optical, nanomaterial-based, and paper-based biosensors could provide rapid naked-eye detection of sweet potato viruses without the need for sophisticated laboratory equipment. Aptamer-based detection systems may eventually replace antibody-dependent ELISA platforms because of their superior stability, low production cost, and high sensitivity. Integration of biosensors with microfluidics and smartphone-based signal processing may further facilitate real-time monitoring and digital disease surveillance [163,165]. These technologies may improve the detection of infections that are difficult to identify using traditional ELISA methods. Isothermal amplification techniques such as LAMP and reverse transcription recombinase polymerase amplification (RT-RPA) are also gaining importance because they eliminate the need for expensive thermal cyclers. Portable devices such as pocket LAMP platforms further enhance the field applicability of LAMP and RT-RPA assays by enabling reliable diagnostics without dependence on mains electricity [102,103]. Also, the cost analysis showed that pocket devices provide a more economical platform which increases accessibility to people. However, their application in remote areas with limited or no internet connectivity may be constrained. Although results can still be displayed in real time on a mobile device, cloud-based data storage and synchronization may be delayed or unavailable [104]. Environmental conditions, such as temperature and humidity, can reduce the sensitivity and specificity of the assays, increasing the risk of false-positive and false-negative results [189].

AI and machine learning are also expected to play an increasingly important role in nondestructive virus diagnosis. Deep learning algorithms integrated with hyperspectral imaging, UAV-based monitoring systems, and smartphone applications could provide early detection of viral infections before visible symptom expression. Future studies should focus on developing large, annotated datasets of sweet potato viral symptoms under diverse environmental conditions to improve algorithm robustness and minimize false-positive predictions. Combining AI-based phenotyping with molecular confirmation platforms may establish highly efficient precision agriculture systems for disease management [49]. Table 3 presents recommended diagnostic technologies for sweet potato virus detection across different application scenarios, highlighting suitable methods based on field conditions, resource availability, and diagnostic requirements.

The selection of diagnostic methods should also consider their suitability for certification programs and epidemiological surveillance. For virus-free planting material certification, highly sensitive and specific techniques such as qRT-PCR, multiplex qRT-PCR, ddPCR, and CRISPR-based assays are particularly valuable because they can detect latent and low-titer infections that may escape conventional testing. In contrast, large-scale epidemiological surveillance benefits from high-throughput and broad-spectrum approaches, including HTS, nanopore sequencing, and multiplex molecular assays, which enable the simultaneous detection of multiple known and emerging viruses across diverse geographical regions.

Future research should prioritize the standardization and validation of molecular diagnostic protocols across laboratories and geographic regions. Variability in nucleic acid extraction methods, reference genes, primer specificity, and sample quality currently limits reproducibility among studies. Establishing internationally harmonized protocols and open-access genomic databases for sweet potato viruses would improve global surveillance and facilitate rapid identification of novel strains and recombinants. In addition, there is an urgent need to strengthen virus indexing, certification systems, and clean seed programs using advanced molecular diagnostics. Since sweet potato is vegetatively propagated, the accumulation and dissemination of latent viruses through planting materials remain major challenges. Integrating rapid molecular detection with thermotherapy, meristem culture, and virus elimination strategies will be essential for sustainable production systems and safe germplasm exchange worldwide [4,16,190].

## 12. Conclusions

The past decades have observed remarkable advances in the molecular detection methods available for sweet potato virus diagnosis, due to their sensitivity and dependability. Conventional RT-PCR, multiplex assays, and qRT-PCR remain the workhorses of routine diagnostic programs, offering proven sensitivity and specificity for known virus targets. For on-site, equipment-free diagnostics, with demonstrated suitability for sweet potato quality assurance in field conditions, isothermal amplification technologies—particularly LAMP and RPA—can be emerged as transformative platforms. Small RNA deep sequencing, HTS, and NGS-based viral metagenomics have fundamentally expanded our knowledge of the sweet potato virome and enabled the simultaneous detection of numerous known and unknown viruses, establishing these approaches as crucial tools for both virology research and sensitive virus screening. In recent years, CRISPR-Cas-based diagnostic systems have shown great potential for point-of-care pathogen detection due to their high sensitivity, specificity, and field deployability; however, no CRISPR-Cas-based diagnostic platform has yet been established for sweet potato viruses. Additionally, a substantial gap remains between laboratory-based technological developments and reliable field-level diagnostics. Several challenges continue to hinder accurate virus detection in sweet potato, including the underground nature of edible storage roots, low or uneven virus distribution in leaf, stem and root, changing virus transmission patterns, and interference caused by complex plant extracts. In particular, protein adsorption, matrix-associated inhibitors from sweet potato root tissues, and viral infection of storage roots can reduce sensing efficiency, often requiring labor-intensive pre-treatment procedures and the development of more robust diagnostic systems. Addressing these limitations is essential for achieving accurate, practical, and continuous virus surveillance in sweet potato production systems. Future efforts should focus on developing affordable, multiplex, and field-ready diagnostic platforms that integrate isothermal amplification, CRISPR-based detection, lateral flow assays, and digital reporting into portable and user-friendly systems. Such integrated technologies will be critical for virus-free seed production, quarantine management, resistance breeding, and sustainable disease surveillance, particularly in low-resource regions where sweet potato is vital for food security.

## Figures and Tables

**Table 1:** Major Viruses Infecting Sweet Potato, Their Classification, Transmission, Symptoms, and Economic Importance.

Virus	Family/Genus and Genome Type	Main Vector/Spread	Typical Symptoms	Economic Importance	Major Localization	References
SPFMV	*Potyviridae*, *Potyvirus*, ssRNA, positive-sense	Aphids, especially *Myzus persicae* and *Aphis gossypii*; infected vines	Often symptomless; mild leaf spots, light green vein patterns; sometimes cracking or internal corkiness in storage roots	Most widespread sweet potato virus; usually mild alone, severe in mixed infections	Worldwide	[15,24,25,26]
SPVG	*Potyviridae*, *Potyvirus*, *Potyvirus gebatatae* ssRNA, positive-sense	Aphids; infected planting material	Mild mottling, vein yellowing, ring spots, leaf distortion	Contributes to severe disease in mixed infections	Asia, Africa, Egypt, Barbados USA, Peru	[22,27,28]
SPVC	*Potyviridae*, *Potyvirus*, *Potyvirus cebatatae*, ssRNA, positive-sense	Aphids; infected planting material	Mottling, vein banding, leaf deformation	Important in potyvirus complex	Asia, Africa, Egypt, USA	[22,27,28]
SPV2	*Potyviridae*, *Potyvirus*, *Potyvirus duobatatae*, ssRNA, positive-sense	Aphids; infected planting material	Chlorotic spots, diffuse mottling	Severe in mixed/triple infections, especially with SPCSV, SPVG	Taiwan, USA, China, South Africa, Portugal, Australia, Spain, Barbados	[22,27,29]
SPLV	*Potyviridae*, *Potyvirus*, ssRNA, positive-sense	Aphids; infected planting material	Very mild or no symptoms	Widespread; adds to viral burden in mixed infections	Asia, Africa, India, Egypt	[30,31]
SPCSV	*Closteroviridae*, *Crinivirus*, ssRNA, positive-sense	Whitefly *Bemisia tabaci*	Vein clearing, chlorosis, leaf purpling, stunting	Very destructive; suppresses RNA silencing via RNase3 and worsens co-infections	Worldwide	[14,25,32]
SPLCV and related sweepoviruses	*Geminiviridae*, *Begomovirus*, ssDNA, monopartite	Whiteflies	Leaf curl, chlorotic spots, leaf deformation, reduced leaf size, stunting	Can reduce yield even when foliar symptoms are weak or absent	USA, China, Taiwan, Japan, Korea, Europe, Africa	[25,33,34]
Sweet potato chlorotic fleck virus	*Betaflexiviridae*, *Carlaviridae*, *ssRNA*	Usually via vegetative material; mixed infections	Chlorotic flecks, leaf mottling, reduced vigor	Less harmful alone, more important in mixed infection	Africa, Asia, South America, New Zealand, Cuba, Pana	[35,36,37]
Cucumber mosaic virus (CMV)	*Bromoviridae*, *Cucumovirus*, *ssRNA*, positive-sense	Aphids and other general vectors	Mosaic, chlorosis, leaf distortion, stunting	Not sweet potato-specific, but can worsen disease complexes	Israel, Egypt, Kenya, Uganda, Japan, South Africa, N. Zealand (worldwide in other crops)	[38,39]
Sweet potato pakakuy virus (SPPV)	*Caulimoviridae*, *Badnavirus*, DNA virus	Infected planting material	Often mild or variable symptoms	Part of the sweet potato virome	Multiple continents in worldwide	[40,41]
Sweet potato mastrevirus 1 (SPSMV-1)	*Geminiviridae*, *Mastrevirus*, *ssDNA*	Whitefly-associated/plant material	Usually symptomless	Still important in the overall virome and mixed infections	Brazil, China, Kenya, Korea, Peru, Taiwan, Tanzania, Uruguay, USA, and Spain.	[40,42]

**Table 2:** Integrated Molecular and Emerging Technologies for Sweet Potato Virus Detection.

Method	Target	Sensitivity	Detection Limit	Field Deployability	Cost, Time	Key Application	Reference
Symptom-based diagnosis	Visual symptoms	Low	No reliable limit reported	High	Very low, no fixed time	Preliminary field inspection	[8,102]
Biological indexing	Host response/inoculation	Moderate	No fixed detection limit	Low	Moderate, Several Weeks	Confirmation of latent infection	[46,47,102]
Electron microscopy	Virus particles	Moderate	10^5^–10^6^ particles/mL	Low	Low, Same-day after sample prep	Visualizing virions and structure	[45,175,176]
ELISA/DAS-ELISA/Dot-ELISA	Viral antigens	Moderate	0.1–10 ng/mL	Moderate	Low–moderate, 2–6 h	Routine screening and indexing	[57,60,61,102,175]
Immunocapture PCR/ELISA-qRT-PCR	Antigen + nucleic acid	High	1–10 copies/µL	Low	Moderate–high, 2–6 h	Sensitive confirmation of low-titer infections	[29,61,64]
Single RT-PCR	Viral RNA	High	1–10 copies/µL	Low	Moderate, 2–4 h	Specific detection of one virus	[25,77,177]
Multiplex RT-PCR	Multiple viral RNAs	High	1–10 copies/µL	Low	Moderate, 1–2 h	Mixed-virus diagnostics in sweet potato	[56,79,82,175]
qRT-PCR	Viral RNA load	Very high	1–10 copies/µL	Low	Moderate–high, 1–2 h	Quantification and confirmation	[31,90,91]
Aptamer-based detection	Viral proteins/targets	High	0.43 ng/mL to picomolar range	Moderate	Moderate, minutes to few hours	Specific recognition and biosensing	[169,170,178]
Rolling circle amplification	Circular DNA viruses	High	Moderate, ~1.3 fmol	Moderate	Moderate, 1 h	Badnavirus and DNA virus detection	[114,116,179]
LAMP	Viral DNA/RNA	High	Moderate, 10 copies/reaction.	High	Low, 5–45 min	Rapid on-site screening	[104,105]
RPA	Viral DNA/RNA	High	Moderate, 10^2^ copies/μL	Very high	Low–moderate, 1 h	Portable field detection	[122,123,180]
Small RNA deep sequencing	Viral small RNAs	Very high	High, No fixed numeric detection limit	Low	High, Several days	Virome profiling and discovery	[54,154,156]
HTS	Whole virome	Very high	Very high, No fixed numeric detection limit	Low	High, Several days	Unknown virus discovery and mixed infections	[140,181]
Nanopore sequencing	Whole genomes/real-time reads	High	High, No fixed numeric detection limit	Moderate	Moderate–high, 8 h to 1 day	Rapid diagnostics and genome recovery	[148,149]
Biosensing technology	Viral antigens/biomarkers	High	Moderate, variable (about 25 fg/μL)	High	Moderate, few minutes	Rapid field diagnostics assets	[162,163,182]
AI-based imaging	Symptom/spectral patterns	Moderate–high	Very high, variable (5 viral copies/μL)	High	Moderate, few minutes	Large-scale field screening assets.	[8,49,183]
CRISPR/Cas detection	Viral nucleic acids	Very high	Moderate, <10 copies/µL	High	Moderate, 30–60 min	Rapid, highly specific point-of-care detection	[130,181]

**Table 3:** Recommended technology selection for sweet potato virus detection under different application scenarios.

Scenario	Recommended Technology	Justification
Field surveillance	AI-based imaging, LAMP, RPA, biosensing technology, CRISPR/Cas	Rapid, non-destructive, on-site screening, portable readouts with imaging, non-destructive monitoring and molecular confirmation
Planting materials & Seed certification	Multiplex RT-PCR, qRT-PCR, ELISA/DAS-ELISA, hybridization/qRT-PCR workflows	High sensitivity; rapid turnaround.
Virus discovery	HTS, small RNA deep sequencing, Nanopore sequencing	Unbiased detection, resolving mixed infections, and providing genome-level information
Routine screening	ELISA/DAS-ELISA/Dot-ELISA, single RT-PCR, multiplex RT-PCR	Affordable and practical processing of many samples
Early detection	qRT-PCR, RPA, LAMP, CRISPR/Cas, aptamer-based detection	Pre-symptomatic detection of low-titer infections, high specificity and rapid readouts
Gene level detection & analysis	qRT-PCR, single RT-PCR, multiplex RT-PCR, small RNA deep sequencing	Transcript-level studies, viral load estimation, and molecular characterization of infection responses
Resource-limited	LAMP, RPA, Dot-ELISA, biosensing technology, AI-based imaging	Minimal equipment; low cost and easier to deploy at field
Mixed infections detedtion	Multiplex RT-PCR, qRT-PCR panels, HTS, Nanopore sequencing	Comprehensive detection of multiple viruses simultaneously
Real-time monitoring	Nanopore sequencing, AI-based imaging, biosensing technology, portable CRISPR/Cas	Portable; rapid analysis, near-real-time detection, field surveillance and outbreak tracking
Availability& Cost effectivity	ELISA, Dot-ELISA, LAMP, RPA	High accessibility and cost-effective options for routine use and field deployment

## Data Availability

The data presented in this study are available on request from the corresponding author.
